# Проблема применения гормональной терапии, направленной на коррекцию пола, трансгендерными лицами по собственной инициативе

**DOI:** 10.14341/probl12806

**Published:** 2022-04-30

**Authors:** Е. В. Макарова, Н. В. Соловьева, С. А. Кременицкая

**Affiliations:** Научный центр персонализированной медицины; Национальный научно-исследовательский институт общественного здоровья им. Н.А. Семашко; Научный центр персонализированной медицины; Научный центр персонализированной медицины

**Keywords:** трансгендерное здоровье, заместительная гормональная терапия, тестостерон, эстрогены, маскулинизация, феминизация

## Abstract

**ВВЕДЕНИЕ:**

ВВЕДЕНИЕ. В последние годы значимо возрос запрос на феминизирующую и маскулинизирующую гормональную терапию среди трансгендерных людей во всем мире. В России отсутствуют подобные исследования, и количество медицинской информации о популяции трансгендерных лиц ограничено.

**ЦЕЛЬ:**

ЦЕЛЬ. Оценить количество трансгендерных пациентов, которые принимают гормональную терапию без назначения врача, а также дать характеристику используемым ими препаратам.

**МАТЕРИАЛЫ И МЕТОДЫ:**

МАТЕРИАЛЫ И МЕТОДЫ. В анализ были включены данные 1117 трансгендерных пациентов (44,01% (n=515) из них были трансгендерными женщинами, 55,99% (n=630) были трансгендерными мужчинами), обратившихся в «Научный центр персонализированной медицины» с целью получения медицинской помощи.

**РЕЗУЛЬТАТЫ:**

РЕЗУЛЬТАТЫ. На момент обращения за медицинской помощью половина трансгендерных пациентов (53,6%) уже принимали гормональные препараты — чаще трансгендерные женщины (76,7%), реже трансгендерные мужчины (32,3%). К эндокринологам за назначением терапии при этом обращались только 8,6% из них. Многие пациенты применяют нелицензированные средства, используют нерациональные схемы и комбинации, зачастую встречается передозировка.

**ЗАКЛЮЧЕНИЕ:**

ЗАКЛЮЧЕНИЕ. Значительная часть трансгендерных людей начинают применять заместительную гормональную терапию по собственной инициативе, без контроля врачей. Решением данной проблемы могли бы быть повышение уровня знаний врачей и пациентов для формирования доверительной среды и продуктивного взаимодействия между терапевтами, эндокринологами и трансгендерными людьми, а также организация консультативных центров в рамках государственных медицинских учреждений.

## Abstract (en)

**BACKGROUND:**

BACKGROUND: In recent years, the demand for feminizing and masculinizing hormone therapy has increased significantly among transgender people around the world. There are no such studies in Russia and the amount of medical information on the transgender population is limited.

**AIM:**

AIM: to assess the number of transgender patients who take hormone therapy without a doctor’s prescription and to characterize the medicines they use.

**MATERIALS AND METHODS:**

MATERIALS AND METHODS: data from 1117 transgender patients were included in to analysis: 44.01% (n=515) of them were trans women, 55.99% (n=630) were trans men. Patients applied to the “Scientific Center for Personalized Medicine” in order to receive medical care.

**RESULTS:**

RESULTS: Half of transgender people (53.6%) are already taking hormonal drugs. More often transgender women (76.7%), less often transgender men (32.3%). In this case, only 8.6% turned to endocrinologists for the therapy prescription. Many patients used unlicensed drugs, use irrational schemes and combinations, often overdose.

**CONCLUSION:**

CONCLUSION: A significant number of transgender people start using hormone replacement therapy on their own initiative, without the doctor’s supervision. The solution to this problem could be incensement of knowledge level in doctors and patients to create a friendly environment and productive interaction between therapists, endocrinologists and transgender people, as well as the organization of consulting centers within public medical institutions.

## ВВЕДЕНИЕ

В последние годы во всем мире и в Российской Федерации в частности происходит повышение обращаемости за медицинской помощью трансгендерных и гендерно-неконформных людей [1][2]. По оценкам недавнего демографического исследования, в США 0,39% (около миллиона человек) идентифицируют себя как трансгендерные люди [3]. Однако истинная распространенность может быть значительно выше. Исследование GIRES указывает цифру 1% среди взрослого населения Великобритании [4].

Согласно определению Всемирной Профессиональной Ассоциации Трансгендерного Здоровья (WPATH), гендерная дисфория (ГД; англ. gender dysphoria) — это дискомфорт или дистресс, вызванный расхождением между гендерной идентичностью человека и приписанным ему при рождении полом, связанной с этим гендерной ролью, первичными и вторичными половыми признаками [5].

Как и любой хронический стресс, дисфория значимо снижает качество жизни (КЖ) людей и уровень их социализации [2–4]. Для снижения ГД таким пациентам показано гендерно-аффирмативное лечение, а именно: хирургическая коррекция и заместительная гормональная терапия (ЗГТ), что помогает привести тело человека в желаемый вид и достичь гармонии с собственным полом [3–5]. Процесс активных изменений у таких лиц принято называть «переходом» (англ. transition). Накоплен достаточный объем научных данных, которые демонстрируют, что гендерно-аффирмативные процедуры улучшают качество жизни транс-пациентов, снижают тревогу, депрессию, повышают социальную и физическую активность, снижают частоту попыток суицидов в этой популяции [3][5], которая в случае неоказания помощи достигает 41% [6].

Отметим, что термин «транссексуализм», фигурирующий в МКБ-10 под кодом F64.0, на сегодняшний день считается устаревшим и стигматизирующим [7]. В новой, 11-й версии МКБ данный диагноз исключен из классификации, вместо него добавлено понятие «гендерное несоответствие» (англ. gender incongruence), которое отнесено в раздел «состояния, связанные с сексуальным здоровьем» [7].

Поскольку запрос на гендерно-аффирмативную медицинскую помощь и коррекцию ЗГТ с каждым годом только возрастает [2–4], врачебное сообщество, и в первую очередь эндокринологи, должны быть готовы работать с трансгендерными людьми, которые, безусловно, имеют свое право на здоровье наравне с остальными гражданами.

На сегодняшний день дефицит информации и среди медицинского персонала, и среди самих трансгендерных людей формирует серьезный барьер, снижающий доступность любой медицинской помощи в этой популяции. Боясь столкнуться с некорректным обращением в условиях государственной системы здравоохранения, многие трансгендерные люди откладывают поход к врачу, в том числе по вопросам, не связанным с «переходом». С другой стороны, даже толерантные доктора, сталкиваясь с такими пациентами, могут оказаться недостаточно информированы для оказания помощи, поскольку данная тема не входит в программы образования в медицинских вузах [8].

Согласно приказу Министерства здравоохранения Российской Федерации от 23.10.2017 № 850н «Об утверждении формы и порядка выдачи медицинской организацией документа об изменении пола» от 2 февраля 2018 г., получить разрешение на смену гендерного маркера в документах и дальнейшие аффирмативные процедуры возможно по достижении 18 лет, после утверждения диагноза F64.0 комиссией, состоящей из психиатра, сексолога и психолога [9].

В идеале маршрут трансгендерного пациента начинается с комиссии психиатров, которая исключает тяжелые психические расстройства и корректирует сопутствующие коморбидные состояния, после чего пациент обращается к эндокринологам и хирургам. Однако реальная картина может значительно отличаться от формального порядка вещей.

Трансгендерный человек живет с сильной дисфорией и испытывает желание привести внешний облик в соответствие с собственными представлениями о себе. Однако при низком доверии к системе здравоохранения и недостаточной квалификации врачей такие люди имеют ограниченный доступ к медицинской помощи.

Оказавшись в такой ситуации, многие трансгендерные люди прибегают к началу гормонотерапии по собственной инициативе для снижения дисфории. В таком сценарии старт терапии происходит без назначений врача, на основании информации из интернета или советов друзей, из соображений финансовой выгоды, но не безопасности и рациональности. Выбранные для терапии препараты могут приобретаться полулегально, не иметь надлежащей сертификации и относятся к категории биологически активных добавок (БАД) [10].

Известно, что длительное нерациональное применение препаратов эстрадиола может приводить к гиперкоагуляции и развитию тромбозов [11], отекам [12], повышению пролактина и вторичным аденомам гипофиза [13], спорным остается вопрос о развитии онкологических заболеваний [14], в том числе рака груди [15][16]. Передозировка препаратами тестостерона, в свою очередь, ассоциирована с повышением гематокрита и тромбокрита [17], артериальной гипертонией [18], инсулинорезистентностью [19], акне [20], развитием поликистоза яичников и рака шейки матки [12][14]. С учетом всего вышесказанного, не вызывает сомнений опасность использования гормональной терапии по собственной инициативе, поскольку отсутствие надлежащего врачебного контроля несет серьезный риск для здоровья молодых работоспособных людей.

Проблема использования маскулинизирующей и феминизирующей ЗГТ по своей инициативе хорошо известна во всем мире, однако исследований, подробно освещающих этот вопрос, немного. В американском исследовании Xavier J. et al. (2000) говорят о том, что 58% из общей выборки трансгендерных пациентов когда-либо применяли гормональные препараты без назначения врача [21], среди них почти 60% трансгендерных женщин (male-to-female, MtF) и 22% трансгендерных мужчин (female-to-male, FtM) [22]. В исследовании, проведенном в Чикаго, 71% MtF сообщили о самостоятельном составлении схемы гормональной терапии, а в Сан-Франциско 29% MtF и 3% FtM сообщили об использовании ЗГТ по собственной инициативе за последние 6 мес [23]. Исследование, куда были включены MtF, проживающие в Нью-Йорке, показало, что 23% принимали терапию, составленную без участия врача [24]. Rotondi N.K. et al. (2013) изучили популяцию трансгендерных лиц в Канаде [8], куда вошли 433 пациента (47,2% MtF, 52,8 FtM), 26,8% из них когда-либо принимали ЗГТ по собственной инициативе. Mepham N. et al. (2014) было проведено исследование с целью анализа источников получения гормональных средств трансгендерными лицами в Великобритании. По их данным, 69% пациентов приобретали препараты через Интернет без наличия рецепта, 22% получали рецепты в частных клиниках, 6% – от друзей и только 3% – от своего терапевта (врача общей практики) [10].

В России отсутствуют подобные исследования, и количество медицинской информации о популяции трансгендерных лиц ограничено. При этом растущий запрос на гендерно-аффирмативные процедуры требует изучения проблемы для лучшего понимания, каким образом система здравоохранения может помочь этим пациентам.

## ЦЕЛЬ ИССЛЕДОВАНИЯ

Оценить количество трансгендерных пациентов, которые принимают гормональную терапию без назначения врача, а также дать характеристику используемым ими препаратам.

## МАТЕРИАЛЫ И МЕТОДЫ

Был проведен ретроспективный анализ карт пациентов с диагнозом «транссексуализм» (F64.0), обратившихся в «Научный центр персонализированной медицины» (НЦПМ) г. Москвы с января 2014 по апрель 2021 гг. с целью получения медицинской помощи.

Дизайн исследования: ретроспективное описательное исследование одномоментного среза.

Переменные. Были проанализированы общее количество обращений, количество обратившихся пациентов, возраст обращения за аффирмативной помощью, данные анамнеза жизни (возраст осознания трансгендерности, принятия себя трансгендерным человеком, возраст первых шагов по смене пола), данные фармакологического анамнеза (факт приема феминизирующей или маскулинизирующей гормональной терапии до обращения в клинику, срок приема препаратов, факт обращения к эндокринологам за назначением терапии, препараты и их дозировки на момент обращения в клинику), наличие осложнений на фоне гормональной терапии.

Характеристики группы. Средний возраст обращения составил 26,5 года (от 15 до 65 лет). Для MtF: 28,3 года (от 15 до 65 лет), для FtM: 25,2 года (от 16 до 52 лет). Ощущения себя лицом не своего пола либо отрицание пола, приписанного при рождении, большинство пациентов отмечали с детства: в среднем с 9,5 года (от 2 до 45 лет). Эти цифры не совпадают с классическими представлениями отечественной психиатрии, где возраст осознания обозначен как 3–4 года [25].

Медиана возраста принятия себя трансгендерным человеком приходилась на 18,04 года (от 10 до 64 лет), гораздо позже возраста появления проблем, по мере поступления информации и нарастания ГД (пик которой обычно приходится на пубертат и активные изменения тела). Какие-либо шаги по смене пола реализуются пациентами еще позже, в среднем в 22,5 года (от 12 до 64 лет). Под шагами по смене пола нами подразумевается: каминг-аут перед друзьями, использование соответствующих аксессуаров и одежды, использование в речи желаемых гендерных окончаний и местоимений, выбор имени. Из всех пациентов 64% (n=715) отмечали в течение жизни этапы «переделки себя», а именно попытки жить в социальной роли, соответствующей паспортному полу (табл. 1).

**Table:** Таблица 1. Характеристика пациентовTable 1. Patient characteristics Примечание. Результаты приведены в виде Me [Min; Max].

Параметры	Все включенные в исследование (n=1117)	MtF (n=515)	FtM (n=630)
Средний возраст обращения с целью прохождения комиссии, лет	26,5 [ 15; 65]	28,3 [ 15; 65]	25,2 [ 16; 52]
Осознание себя лицом не своего пола, лет	9,5 [ 2; 45]	10,5 [ 2; 45]	8,8 [ 3; 40]
Принятие себя трансгендерным человеком, лет	18,04 [ 10; 64]	19,5 [ 10; 64]	16,9 [ 10; 40]
Первые шаги по смене пола, лет	22,5 [ 12; 64]	24,2 [ 13; 64]	21,2 [ 12; 47]

Статистический анализ: проводился в программе Statistica 11.0 с использованием методов описательной статистики.

Этическая экспертиза: исследование одобрено на заседании локального этического комитета при НЦПМ от 14.08.2021, протокол №115.

## РЕЗУЛЬТАТЫ

## Число обращений

В анализ были включены данные 1117 трансгендерных пациентов: 44,01% (n=515) из них были трансгендерными женщинами (male-to female, MtF), 55,99% (n=630) были трансгендерными мужчинами (female-to-male, FtM).

За указанный временной период отмечен отчетливый рост количества обращений с целью получения справки. Если в 2014 г. мы имели только 77 обращений, то в 2020 г. эта цифра достигла 237 человек, что говорит о росте +123,2% за 7 лет. Данные о приросте обращений представлены на рисунке 1. Только за период с января по апрель 2021 г. в клинику обратились 90 пациентов.

**Figure:**
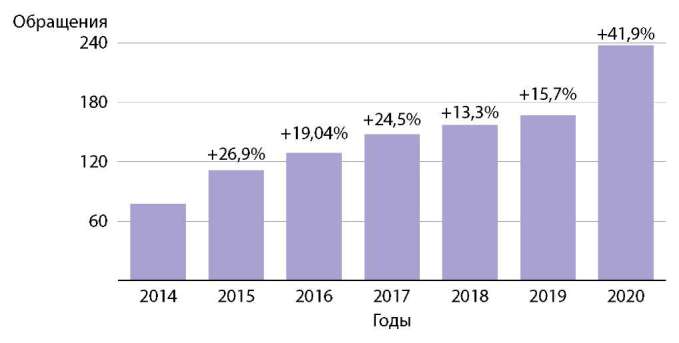
Рисунок 1. Рост числа обращений трансгендерных пациентов по годам.Примечание. Прирост в % указан относительно предыдущего годаFigure 1. Growth in the number of visits of transgender patients by years.Note. Growth in % is indicated relative to the previous year

## Старт гормональной терапии по своей инициативе

При анализе фармакологического анамнеза было выявлено, что среди всех трансгендерных пациентов, обратившихся за получением справки «О половой переориентации» и разрешением на аффирмативные процедуры, 599 человек, или 53,6%, уже принимали заместительную терапию половыми гормонами желаемого пола (рис. 2). Срок терапии составлял от 1 мес до 20 лет, в основном от 2 до 5 лет.

**Figure:**
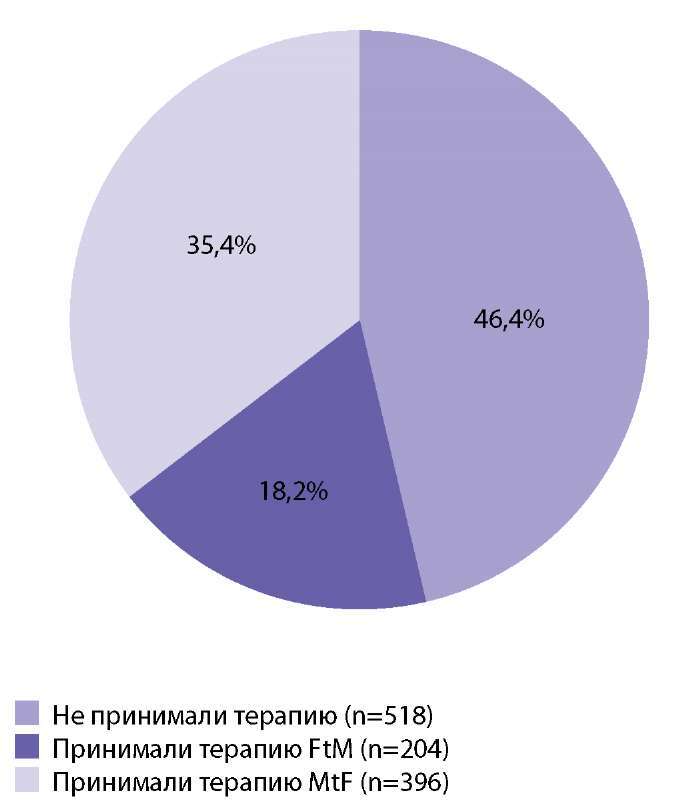
Рисунок 2. Распространенность использования гормональной терапии по собственной инициативе среди трансгендерных людей (n=1117).Figure 2. Prevalence of self-initiated hormone therapy use among transgender people (n=1117).

Феминизирующая терапия была начата у 396 лиц MtF (76,7% всех транс-женщин). Маскулинизирующая терапия была начата у 204 пациентов FtM (32,3% всех транс-мужчин). Подробные данные представлены на рис. 3.

**Figure:**
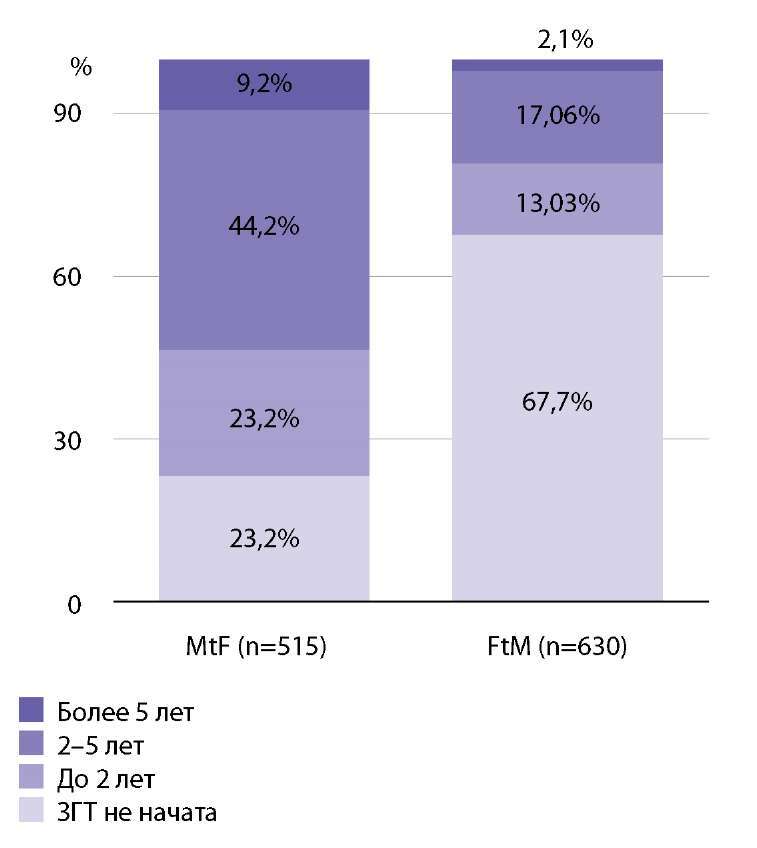
Рисунок 3. Длительность заместительной гормональной терапии (n=1117)Figure 3. Duration of hormone replacement therapy (n=1117)

Длительность гормональной терапии до прохождения комиссии для MtF в среднем составила 2,2 года, а для FtM — 0,7 года.

Среди трансгендерных лиц, начавших принимавших гормональные препараты, до прохождения комиссии к эндокринологу за составлением терапии обращались только 8,6%. Соответственно, у 91,4% схема лечения была составлена по примеру друзей либо на основании неофициальной информации, представленной в интернет-сообществах, на зарубежных и российских сайтах. Только 15,4% отметили, что перед стартом лечения по собственной инициативе они ознакомились с «Международными медицинскими стандартами помощи трансгендерным людям» [5], статьями в Pubmed, международными рекомендациями или другими серьезными научными источниками.

## Препараты для заместительной гормональной терапии

В качестве препаратов для маскулинизирующей гормональной терапии в группе FtM применялись следующие лекарственные средства:

Лекарственные средства для феминизирующей гормональной терапии представляли значительно более обширный ассортимент. В 97,5% случаев транс-женщины принимали эстрогены:

Ряд пациенток (9,71%), кроме эстрогенов или комбинированных препаратов, принимали прогестерон — как правило, капсулы американских производителей.

Если препарат, выбранный для феминизирующей гормональной терапии, не содержал ципротерона ацетат, то в схему обычно дополнительно включались антиандрогены, что соответствует международным стандартам:

Стоит отметить, что схемы феминизирующей терапии характеризовались большим разнообразием, комбинацией нескольких эстрогеновых и нескольких антиандрогенных препаратов, что приводило к полипрагмазии.

Немногие из пациенток MtF использовали 1 препарат. Как правило, это были два и более (до 6) веществ, обладающих гормональной активностью.

## Осложнения в ходе заместительной гормональной терапии

Данное исследование являлось ретроспективным, и оценка частоты побочных эффектов на фоне гормональной терапии по собственной инициативе не входила в задачи работы, однако авторы считают необходимым на основе своего клинического опыта обратить внимание на наиболее частые явления, зафиксированные у наших пациентов из описанной выше выборки.

## ДИСКУССИЯ

ГД и трансгендерность не являются прихотью человека, а представляют серьезную медицинскую проблему [1]. Наш клинический опыт общения с этими людьми подтверждает данную позицию. Обеспечение доступности и инклюзивности медицинской помощи трансгендерным лицам имеет важное значение для поддержания благополучия, здоровья, трудоспособности и социальной активности этой немаленькой популяции [28].

На сегодняшний день в РФ нет установленного порядка оказания медицинской помощи трансгендерным людям, однако именно эндокринологи зачастую оказываются первыми специалистами, к которым обращаются эти пациенты за консультацией с целью составления ЗГТ, коррекции дозы гормональных препаратов, скрининга на предмет развития осложнений. Тем не менее любой врач может столкнуться в своей практике с такими людьми.

Хотя на сегодняшний день в России программа медицинских вузов не подразумевает изучение вопросов трансгендерного здоровья, при участии А.Ю. Бабенко и Д.Д. Исаева были переведены на русский язык «Международные медицинские стандарты помощи трансгендерным людям» 7-го пересмотра [5], которые позволяют получить базовые знания по данному вопросу.

Дефицит знаний отмечают врачи многих стран. К примеру, в работе Johnston C.D. et al. (2017) в ходе опроса студентов было выявлено, что 97% отметили важным приобретение навыков медицинской помощи для трансгендерных пациентов, только 45% имели какие-либо знания в этом вопросе. В исследовании Shires D.A. et al. (2018), куда вошли врачи общей практики, 85,7% респондентов выразили готовность оказывать помощь трансгендерным пациентам, но 48% отмечали препятствия, связанные с низкой компетентностью и недостаточной подготовкой, еще 31% сообщили, что не чувствуют себя достаточно уверенными для оказания рутинной медицинской помощи трансгендерным пациентам [29].

Полученные нами данные о частоте применения ЗГТ по собственной инициативе среди российских трансгендерных лиц сопоставимы с цифрами зарубежных коллег [10][12][22][23]. В отдельных регионах США цифры ниже, вероятно, за счет лучшей организации медицинской помощи этой категории пациентов [24]. Аналогичные результаты получены о более частом применении гормональных средств лицами MtF.

Наше исследование — первая российская работа по изучению данного вопроса, а также охватывает значимо большую выборку трансгендерных людей в сравнении с иностранными публикациями. Зарубежными коллегами также не было детально изучено, какие препараты используются трансгендерными людьми по собственной инициативе.

Ограничениями работы является то, что мы анализировали данные ретроспективно и только среди пациентов, обратившихся в частный медицинский центр. Таким образом, это были люди с определенным уровнем достатка и мотивации для прохождения гендерно-аффирмативных процедур. Истинное число людей, использующих гормональную терапию с целью маскулинизации или феминизации без назначений врача, может быть значительно выше.

В нашей выборке подавляющее большинство трансгендерных пациентов на момент обращения в НЦПМ уже жили в состоянии дисфории достаточно длительно. Решение совершить «переход» не принималось спонтанно, а постепенно формировалось в течение нескольких лет. Из всей группы 86,96% на момент обращения уже жили в социальной роли желаемого гендера от полугода до 30 лет.

Имеющийся перевес транс-женщин над транс-мужчинами, начавших прием ЗГТ без назначения врача, объясняется доступностью препаратов эстрогенов и антиандрогенов, которые возможно приобрести без рецепта, тогда как инъекционные препараты тестостерона отпускаются строго по рецептам формы 148-1/e-88. Подобную картину описывают и зарубежные коллеги.

Невозможность купить препараты официальным путем приводит к использованию нелицензированных препаратов. Опасения вызывают применение БАДов, использование инъекционных форм незарегистрированных ЛС, не имеющих соответствующей сертификации и доказательной базы.

Отдельное внимание хочется уделить тому, что в интернете возможно приобрести форму выпуска смесей эфиров тестостерона во флаконах по 10 мл. Считая это более экономичным, ряд пациентов используют такие флаконы как многоразовые, вводя по 1 мл на одну инъекцию до 10 раз, при этом не заботясь о герметичности хранения и возможном снижении активности действующего вещества.

Решением данной проблемы могли бы быть повышение уровня знаний врачей и пациентов для формирования доверительной среды и продуктивного взаимодействия между терапевтами, эндокринологами и трансгендерными людьми, а также организация консультативных центров в рамках государственных медицинских учреждений.

К сожалению, основная глобальная проблема — это серьезный дефицит качественных длительных исследований по влиянию ЗГТ на состояние здоровья [1][12][29]. К примеру, среди специалистов нет единого мнения о предпочтительных дозах гормональных препаратов, объемах оперативного лечения и постоперационной коррекции терапии. Не меньшего внимания заслуживают такие моменты, как степень удовлетворенности аффирмативными процедурами и случаи «обратного перехода». Трансгендерное здоровье — это большое научное и практическое поле, нуждающееся в изучении, понимании и формировании клинических рекомендаций.

## ВЫВОДЫ

## ДОПОЛНИТЕЛЬНАЯ ИНФОРМАЦИЯ

Источники финансирования. Работа выполнена по инициативе авторов без привлечения финансирования.

Конфликт интересов. Авторы декларируют отсутствие явных и потенциальных конфликтов интересов, связанных с содержанием настоящей статьи.

Участие авторов. Макарова Е.В. — сбор данных, написание текста статьи; Соловьева Н.В. — концепция исследования, сбор материала; Кременицкая С.А. — сбор материала, обработка данных. Все авторы одобрили финальную версию статьи перед публикацией, выразили согласие нести ответственность за все аспекты работы, подразумевающую надлежащее изучение и решение вопросов, связанных с точностью или добросовестностью любой части работы.
